# 218. Development of a Simple Score for Diagnosis Melioidosis

**DOI:** 10.1093/ofid/ofad500.291

**Published:** 2023-11-27

**Authors:** Kanjana Khaejawat, Anupol Panichote, Atibordee Meesing

**Affiliations:** Khon Kaen university, Khonkaen, Khon Kaen, Thailand; Khon Kaen university, Khonkaen, Khon Kaen, Thailand; Khon Kaen university, Khonkaen, Khon Kaen, Thailand

## Abstract

**Background:**

Melioidosis is a common gram-negative bacterial infection in northeastern Thailand. Patients with melioidosis infections often experience severe conditions and high mortality rates. This study aims to develop a clinical prediction model to estimate the risk of melioidosis septicemia.

**Methods:**

This retrospective case-control study included patients with positive hemoculture for *Burkholderia pseudomallei* (BP) and other gram-negative bacteria (*Escherichia coli*, and *Klebsiella pneumoniae*) admitted to Srinagarind Hospital between January 2015 and December 2020. Logistic regression analyses were used to determine the calculation of a score for diagnosing melioidosis infection.

**Results:**

A total of 426 patients with positive hemoculture were included: 132 patients for BP and 294 patients for other gram-negative bacteria. The clinical prediction model for diagnosing melioidosis utilized seven variables: age ≥ 60 years (-2 points), male gender (3 points), duration of symptom onset to hospitalization ≥ 7 days (5 points), occupation as a farmer (3 points), presence of diabetes mellitus (2 points), presence of cancer (-5 points), and platelet count (x 10^9^/L) (200-399.9: 1 point, ≥400: 3 points). The model demonstrated good discrimination (area under the curve: 0.89; 95% CI: 0.86-0.93) and acceptable calibration (Hosmer and Lemeshow goodness of fit test: P-value of 0.252). A cut-off point of the melioidosis score ≥ 5 points (maximum score = 16 and minimum score = -7) resulted in an accuracy of 84.3% (95%CI 80.5-87.6), a sensitivity of 78.8%, and a specificity of 86.7%.

**Figure:**
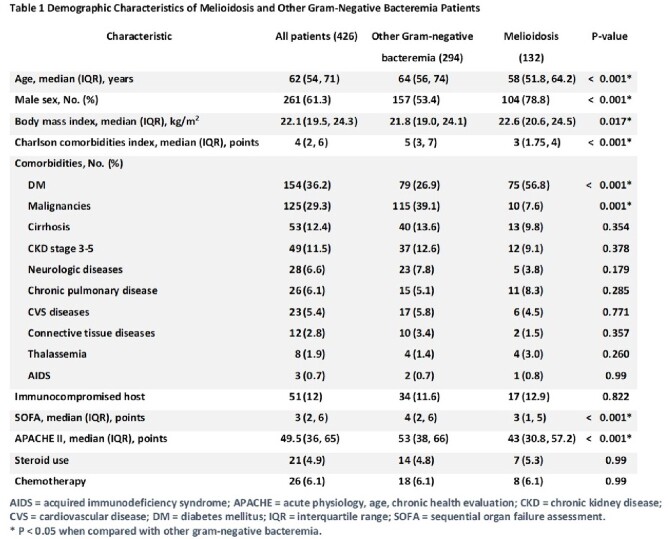

**Figure:**
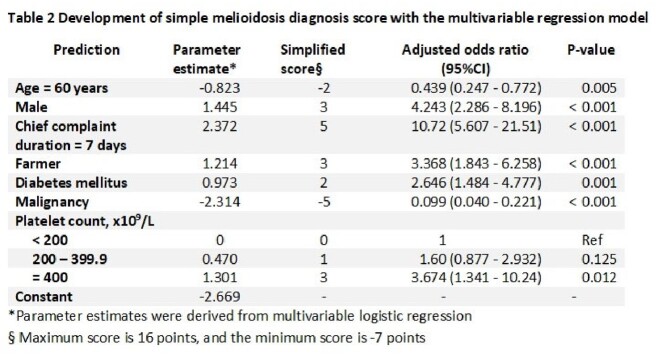

**Figure:**
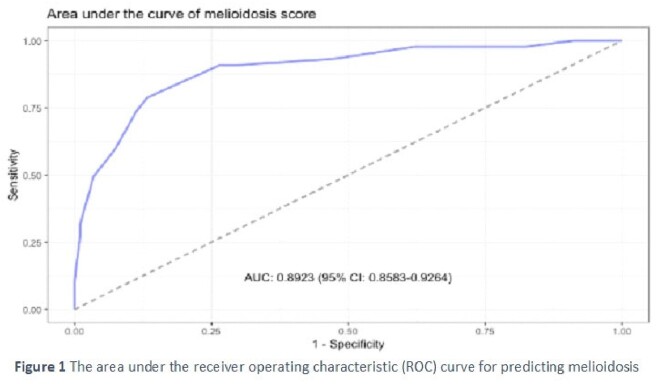

**Conclusion:**

The melioidosis score exhibited high performance and clinical utility in predicting melioidosis infection.

**Disclosures:**

**All Authors**: No reported disclosures

